# Testis-specific serine/threonine protein kinase 4 (Tssk4) phosphorylates Odf2 at Ser-76

**DOI:** 10.1038/srep22861

**Published:** 2016-03-10

**Authors:** Xiaoli Wang, Han Li, Guolong Fu, Yunfu Wang, Shiming Du, Long Yu, Youheng Wei, Shi Chen

**Affiliations:** 1 Key Laboratory of Combinatorial Biosynthesis and Drug Discovery, Ministry of Education, School of Pharmaceutical Sciences, and Medical Research Institute, Wuhan University, Wuhan, 430072, P.R.China; 2 State Key Laboratory of Genetic Engineering, Institute of Genetics, School of Life Sciences, Fudan University, 220 Handan Road, Shanghai 200433, P.R. China; 3 Taihe Hospital, Hubei University of Medicine, Shiyan, Hubei, China

## Abstract

As a member of the testis-specific serine/threonine protein kinase (TSSK) family, Tssk4 is exclusively expressed in the testis and plays an essential role in male fertility. We previously reported that Tssk4 can associate with and phosphorylate Odf2, but the phosphorylation site is still unknown. Here we confirm that the C-terminal region (amino acids 214-638) of Odf2 is required for association with Tssk4. Furthermore, to identify the site at which Tssk4 phosphorylates Odf2, we generated several Odf2 point mutants (Ser/Thr/Lys to Ala) and identified serine 76 of Odf2 as one of the phosphorylation sites. *In vivo*, phosphorylated Odf2 was evaluated in mouse sperm using a specific phospho-Ser-76 Odf2 antibody and LC-MS/MS. These findings are the first to demonstrate the phosphorylation site in Odf2 by Tssk4, providing essential clues regarding the function of Tssk4 in regulating sperm motility and/or structure and thus male fertility.

Reversible protein phosphorylation is critically significant in biology because of its influence on almost every important cellular process throughout phylogeny, including cell growth, cell differentiation, cell cycle, and cell migration^1^. In eukaryotic cells, protein kinases primarily phosphorylate serine (Ser), threonine (Thr), and tyrosine (Tyr) residues by transferring a phosphoryl group to an amino acid side chain^2^. Because phosphorylation is fast, reversible, and often highly specific, it is often employed for temporary modulation of protein function. Phosphorylation modification can alter the global protein structure and the local conformation in a specific peptide motif to change protein-protein interactions and modulate the enzymatic activity of phosphoproteins, resulting in the enhancement or inhibition of protein binding events and protein stability^3^. Over 500 kinases and 100 phosphatases are responsible for catalyzing protein phosphorylation and dephosphorylation and are themselves regulated by phosphorylation, revealing the interconnections among other cellular signaling pathways. Over 250,000 phosphorylation sites have now been mapped in the biological proteome.

The outer dense fibers (ODFs) are prominent sperm tail-specific cytoskeletal structures. They function in forming “9+9+2” structures, together with nine outer doublet microtubules and a central pair of microtubules^4^,^5^,^6^,^7^. In the sperm tail, ODFs are thought to be contractile. In mammalian sperm, the ODFs consist of many proteins with a molecular mass ranging from approximately 11 kDa to approximately 87 kDa^4^,^8^,^9^. The ODF2 protein exhibits several isoforms, with a deduced molecular mass in the range of 60–96 kDa^5^,^10^. Secondary structure prediction indicated that ODF2 is an overall coiled-coil protein^11^. There are two leucine zipper motifs in the C-terminal region of the protein (aa 392–413 and aa 530–551 of rat ODF2), which are responsible for the interaction with the leucine zippers of ODF1 in yeast^5^. Nevertheless, the self-interacting capacity of rat ODF2 is independent of any of the leucine zipper regions^12^. In non-germ cells, ODF2 has been defined as a scaffold protein that is specifically located at the distal/subdistal appendages of mother centrioles where the primary cilia are anchored^13^,^14^. Odf2-deficient mouse E9 cells completely lose the ability to generate primary cilia, indicating that Odf2 is indispensable for the formation of the distal/subdistal appendages of mother centrioles^15^. Odf2^DEx6,7/DEx6,7^ mice display uncoordinated ciliary beating and a coughing/sneezing phenotype because of basal bodies lacking basal feet^16^. Defects due to ciliary abnormalities occur during cell proliferation and are predicted to cause cancer, cystic diseases and fibroses of various sorts, such as polycystic kidney diseases (PKDs)^17^. The transcription factor (TF) Pax6 controls centriole maturation in cortical progenitors through regulating the transcription of the Odf2 gene^18^.

Tssk4 belongs to the testis-specific serine/threonine protein kinase (TSSK) family, whose members play an important role in spermatogenesis and/or spermiogenesis. Thus far, five members of this family have been reported, including Tssk1, Tssk2, Tssk3, Tssk4 (also known as Tssk5), and Tssk6. Due to their specific expression patterns during the processes of spermatogenesis and/or spermiogenesis, their physiological functions were explored by establishing gene knockout mouse models. The *Tssk1* and *Tssk2* double-deletion mouse was haploinsufficient and could not transmit the null genotype^19^. However, another group reported that the double-deletion mouse model displayed male sterility accompanied by chromatid body loss^20^. These discrepancies were ascribed to different genetic backgrounds. Tssk4 knockout male mice exhibited a subfertility phenotype due to seriously decreased sperm motility^21^. Tssk6 deletion resulted in a male infertile phenotype caused by certain morphological defects in the sperm^22^.

We previously reported that Tssk4 is expressed exclusively in the testis and can maintain its kinase activity through autophosphorylation at Thr-197^23^. It was later shown that Tssk4 can lead to cellular apoptosis, depending on its kinase activity^24^. Male Tssk4 knockout mice exhibit an impaired sperm structure and reduced sperm motility, which affects male fertility^21^. Furthermore, Tssk4 can associate with and change the phosphorylation state of Odf2, while ODF2 can potentiate the autophosphorylation activity of Tssk4 at Ser-197^21^.

In the present study, we defined the C-terminal fragment of Odf2, which is essential for the modification of Tssk4, and we then identified Ser-76 as a Tssk4 phosphorylation site in Odf2 both *in vitro* and *in vivo*. The confirmation of the fragment required for the cooperation of two proteins and the phosphorylation site of Odf2 provide crucial clues that deepen our understanding of the roles of Tssk4 and Odf2 in sperm structure and motility and, thus, male fertility^21^.

## Results

### Mutual association between Tssk4 and Odf2 through phosphorylation

**Figure 1:**
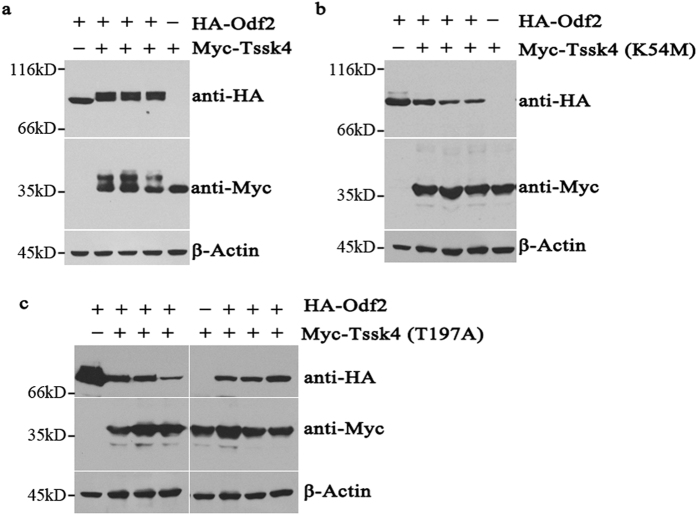
The association between Tssk4 and Odf2. (**a**) Full-length HA-Odf2 was transfected into 293T cells either alone or in combination with Myc-Tssk4, and the electrophoretic migration rates changed for both Odf2 and Tssk4 (2^nd^, 3^rd^, and 4^th^ lanes) when co-expressed compared with the singly transfected Odf2 (1^st^ lane) and Tssk4 (5^th^ lane). (**b**,**c**) Full-length HA-Odf2 was transfected into 293T cells either alone or together with two kinase-dead mutants, including (**b**) Myc-Tssk4 (K54M) and (**c**) Myc-Tssk4 (T197A). The electrophoretic migration rates of the Odf2 and Tssk4 mutants did not change. All the experiments including cell transfection, SDS-PAGE and Western blot were performed under the same experimental conditions. Since there is great molecular weight gap between HA-Odf2 (about 70kD) and Myc-Tssk4 (about 35kD), the blots are cropped to improve the clarity and conciseness of the presentation. The Western blot in all the other figures were showed in the same way.

It has been previously reported that Tssk4 is exclusively expressed in the testis and that male Tssk4 deficiency mice exhibit a subfertile phenotype due to decreased sperm motility and impaired sperm structure^21^. The association between Tssk4 and Odf2 might be the mechanism underlying the subfertility phenotype in the *Tssk4* knockout mouse model^21^. To investigate the connection between Tssk4 and Odf2 in detail, we co-transfected their plasmids into HEK-293T cells and found that the electrophoretic migration rates of both the Tssk4 and Odf2 proteins in sodium dodecyl sulfate (SDS)-polyacrylamide gels were altered (Fig. 1a).

On the one hand, the presence of an Odf2 band with a slower migration rate appeared only when Odf2 was co-transfected with wild-type Tssk4 but not the dead mutant kinase K54M (Fig. 1b) and or the autophosphorylation site mutant T197A (Fig. 1c), implying that Odf2 is a target of the protein kinase Tssk4 and that the phosphorylation modification of Odf2 is dependent on the kinase activity and the autophosphorylation activity of Tssk4. On the other hand, the Tssk4 protein band was also altered, with a slower migration rate when co-expressed with Odf2. This observation has been identified as a phosphorylation modification in our previous work^21^,^23^.

### The Odf2 C-terminus is essential for the phosphorylation state of Tssk4

**Figure 2:**
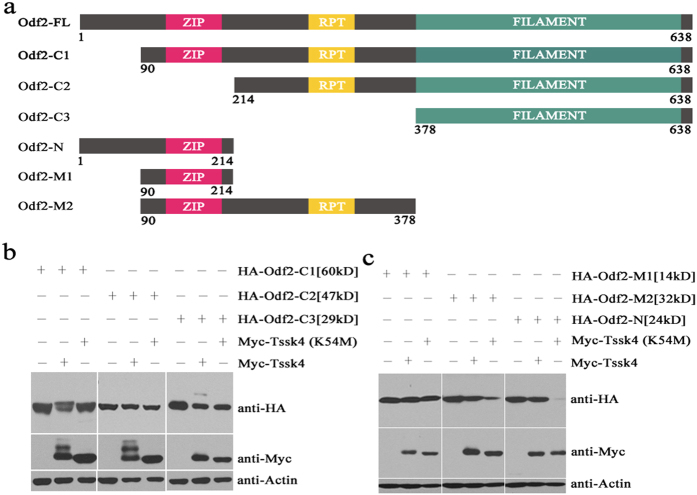
Fragments of Odf2 essential for the modification of Tssk4. (**a**) Full-length Odf2 (HA-Odf2-FL) and six HA-Odf2 truncated constructs according to structural domain prediction using SMART software. (**b**) Myc-Tssk4 was co-expressed either alone or in combination with HA-Odf2-C1, HA-Odf2-C2 and HA-Odf2-C3. Modification of Tssk4 occurred when it was co-transfected with Odf2-C1 and Odf2-C2, and conversely, the electrophoretic migration rate of Odf2-C1 changed when it was co-transfected with Tssk4. (**c**) Myc-Tssk4 was co-expressed either alone or together with HA-Odf2-M1, HA-Odf2-M2 and HA-Odf2-N. There was no modification present on Odf2 or Tssk4.

To identify the essential fragment of Odf2 that is required for altering the phosphorylation state of Tssk4, we generated several truncated constructs of murine Odf2 (GenBank number: NM013615) according to its various functional domains predicted by SMART software (Simple Modular Architecture Research Tool). The computational results revealed 3 major functional domains (Fig. 2a): a leucine zipper (ZIP) domain (amino acids [aa] 119–170); an internal repeat domain, abbreviated as RPT (aa 248–284); and a filament domain (aa 378–631), as well as 4 other disordered/unstructured regions including aa 1–81, aa 89–101, aa 214–234 and aa 310–336 (not shown). The fragments were then sub-cloned into the pCMV-HA vector in frame. According to the functional domains described above, different fragments of Odf2 were sub-cloned, including the C-terminal region, Odf2-C1 (aa 90–638), Odf2-C2 (aa 214–638), and Odf2-C3 (aa 378–638); the N-terminal region, Odf2-N (aa 1–214); and the middle region, Odf2-M1 (aa 90–214) and Odf2-M2 (aa 90–378).

The six truncated constructs were subsequently transfected either alone or together with Tssk4/Tssk4 (K54M). The results revealed that the Odf2-C1 and Odf2-C2 constructs altered the phosphorylation state of Tssk4 (Fig. 2b, left and middle panels), whereas the other four constructs did not (Fig. 2b, right panel, and Fig. 2c), suggesting that RPT and the filament domain of Odf2 are required for its association with Tssk4 and for altering the phosphorylation state of Tssk4.

### Tssk4 phosphorylates Odf2 at Ser-76

**Figure 3:**
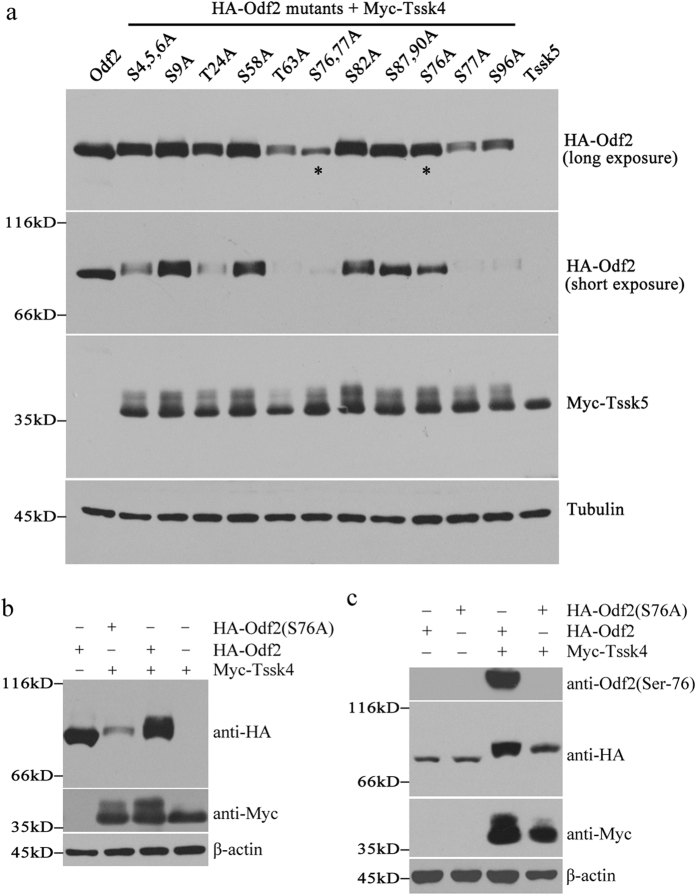
Tssk4 phosphorylates Odf2 at Ser-76. (**a**) A series of Odf2 serine/tyrosine-to-alanine mutants were generated and transfected with Tssk4. The symbol ^*^indicates the possible phosphorylation sites of Odf2, given that there was no obvious change in the migration rate. (**b**) The Odf2 (S76A) mutant displayed no change in electrophoretic motility when co-transfected with Tssk4, implying that serine 76 may be the phosphorylation site catalyzed by Tssk4. (**c**) Tssk4 was co-transfected with wild-type Odf2 and the Odf2 (S76A) mutant, and the phosphorylation band was identified using a specific phospho-Ser-76 Odf2 antibody.

To determine the site in Odf2 that is phosphorylated by Tssk4, a series of computer-predicted serine (Ser) or threonine (Thr) or tyrosine (Tyr) sites in Odf2 were mutated to alanine, either separately or in combination (Fig. 3a). All of these Odf2 mutants were constructed and subsequently transfected into HEK-293T cells, either alone or together with Tssk4. The cell lysates were then subjected to Western blotting to analyze the Odf2 migration rate, which can reflect its phosphorylation status. After a careful analysis of the migration rate of all of the constructed Odf2 mutants, we found that the migration rate of the Odf2-S76A mutant was significantly higher than that of its wild-type counterpart when they were co-transfected with Tssk4 (Fig. 3b). These data indicated that Odf2 might be phosphorylated at Ser-76 by Tssk4. In addition, both Odf2 and its S76A mutant could result in the phosphorylation of Tssk4 *in vitro* (Fig. 3b), implying that the phosphorylation of Tssk4 was independent of the Ser-76 phosphorylation in Odf2. In other words, the association between Odf2 and Tssk4 might be sufficient for the phosphorylation of Tssk4.

To further confirm that the Odf2 Ser-76 phosphorylation site was modified by Tssk4, we generated an anti-phospho-Ser76 Odf2 polyclonal antibody to evaluate the phosphorylation state. The antibody can specifically recognize the phosphorylated Odf2 (Ser-76) protein band (Fig. 3c). When co-expressed with Tssk4, Odf2 was phosphorylated at Ser-76 by Tssk4, while Odf2 transfected alone showed no phosphorylation modification at Ser-76, demonstrating that Tssk4 can phosphorylate Odf2 at Ser-76 (Fig. 3c).

### Identification of serine 76 phosphorylation of Odf2 *in vivo*

**Figure 4:**
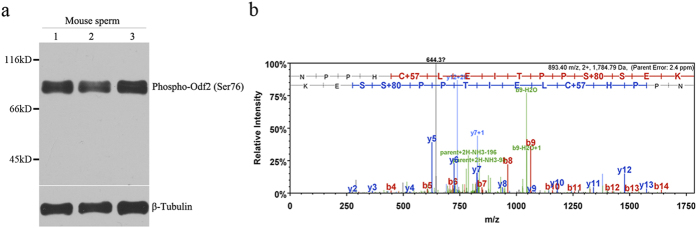
Phosphorylation of Odf2 at Ser-76 *in vivo.* (**a**) Phospho-Ser-76 of Odf2 was detected in mouse sperm lysate using a specific antibody. Three different adult C57BL-6 mouse were used. (**b**) Analysis of the phosphorylation sites via LC-MS/MS. The MS/MS spectrum of the phosphorylated peptide 64-NPPHCLEITPPSSEK-80 displays a series of b- and y-ions, indicating that the underlined serine residue is a putative phosphorylation site.

To determine whether the phosphorylated Ser-76 of Odf2 is present *in vivo*, we performed Western blot analysis using the phospho-Ser76 Odf2 antibody to assess the phosphorylation state in mouse sperm (C57BL-6 strain). As shown in Fig. 4a, a specific band was detected in sperm lysates from three different mouse (experimental triplicates).

The identification of the Ser-76 site phosphorylated by Tssk4 was further verified using LC-MS/MS. A mouse sperm lysate was separated via SDS-PAGE, and the corresponding band of Odf2 was excised and digested with trypsin. LC-MS/MS confirmed the identification of the Odf2 protein (data not shown) and indicated the phosphorylation of the peptide sequence 64-NPPHCLEITPPSSEK-80. The MS/MS spectrum of this phosphorylated peptide is presented in Fig. 4b. The observed fragmentions confirmed the peptide sequence and the presence of one phosphate group on a serine residue, indicating that a phosphorylation site was located at serine residue 76.

**Figure 5:**
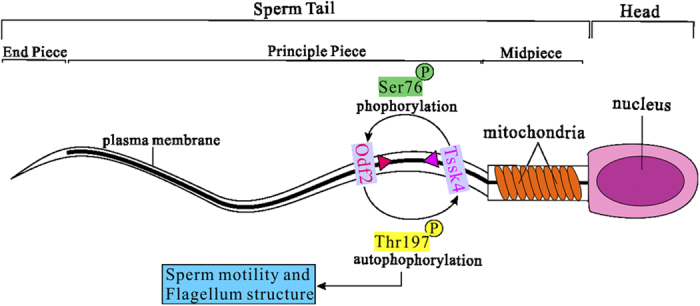
The mutual regulation model between Tssk4 and Odf2 in sperm. Both Tssk4 and Odf2 are located at the midpiece and principle piece of the sperm tail but not at the end piece. Tssk4 phosphorylates Odf2 at Ser-76, which is required for the autophosphorylation modification at Thr-197 in Tssk4. The association of these two proteins and the possible involvement of a signaling pathway might influence sperm motility and/or structure.

In conclusion, as a kinase exclusively expressed in testis, Tssk4 could phosphorylate Odf2 at Ser-76, depending on both its kinase activity and autophosphorylation modification. Meanwhile, Odf2 phosphorylation is required for maintaining the autophosphorylation activity at Thr-197. Figure 5 shows the mutual regulation between Tssk4 and Odf2, which takes part in the regulation of sperm motility and flagellum structure and thus male fertility.

## Discussion

Through phosphorylation site prediction and point mutation technology, we defined the site of Odf2 phosphorylation by Tssk4 both *in vitro* and *in vivo*. However, as the electrophoretic migration rate of the Odf2 (S76A) mutant when it was co-expressed with Tssk4 was slower compared with singly expressed Odf2 (S76A) (Fig. 3c), Ser-76 is not the only phosphorylation site catalyzed by Tssk4. In other words, there should be other sites phosphorylated by Tssk4, including serine, tyrosine and lysine sites. Furthermore, the phosphorylation of Ser-76 in Odf2 is dependent on the kinase activity of Tssk4, given the negative effects of the dead mutant Tssk4 kinase (K54M).

Conversely, the electrophoretic migration rate of Tssk4 changed when it was co-expressed with full-length Odf2 or the Odf2-C1 and Odf2-C2 fragments, indicating that RPT and the filament domain were essential for the modification of Tssk4 and that the type of modification was phosphorylation catalyzed by Tssk4 itself, which was previously reported as auto-phosphorylation activity^24^. Furthermore, the C-terminal region of Odf2 (aa 188–803), corresponding to the HA-Odf2-C2 truncated mutant in our work, was indispensable for ciliogenesis^16^, suggesting dependency on the C-terminal fragment of Odf2 for the modification of Tssk4 and the process of ciliogenesis.

Tssk4 and Odf2 can cooperate to regulate sperm structure and motility, and the deficiency of Tssk4 in a mouse model can lead to disrupted sperm function, thus reducing male fertility^21^. As a kinase, Tssk4 may maintain sperm integrity by dynamically phosphorylating its substrates, including Odf2. In addition, the phosphorylation of Odf2 Ser-76 may also be catalyzed by kinases other than Tssk4. However, we still could not completely clarify that the phosphorylation modification by Tssk4 is directly or indirectly mediated.

Previous studies indicate that ODF functions in maintaining the elastic recoil and passive elastic structures of the sperm flagellum and protecting the sperm flagellum against shear forces during epididymal transport and ejaculation^25^. Because Odf2 is a major component of ODF, the phosphorylation state of Odf2 might affect such functions and could possibly influence sperm motility. Until now, multiple protein kinases have been reported to regulate the activation of sperm tail motility^26^. Although Cdk5-p35^27^,^28^ was identified to cause ODF phosphorylation, no specific kinase that is associated with Odf2 phosphorylation has been discovered. In this study, we provide evidence that Odf2 is a substrate of protein kinase Tssk4 and that the phosphorylation site is Ser-76.

In light of the fact that Odf2 is known to be highly phosphorylated *in vivo*, our discoveries that Tssk4 could phosphorylate Odf2 at Ser-76 and, conversely, that Odf2 phosphorylation is required for the autophosphorylation activity at Thr-197 serve as relevant novel findings that will be useful for further characterization of the role of Tssk4 and phosphorylated Odf2 in sperm. The mutual modification through phosphorylation between Tssk4 and Odf2 and the identification of a functional interaction between these proteins could also provide further insight into signaling pathways that lead to sperm motility and/or structure.

## Materials and Methods

### Plasmids, antibodies and reagents

The pCMV-HA-Odf2 and pCMV-Myc-Tssk4 vectors and the dead mutant Myc-Tssk4 kinase (K54 M) were previously described^21^. The anti-phospho-Odf2 (pSer-76) rabbit polyclonal antibody was custom-made by AbMax Biotechnology Co. Ltd. (Beijing, China) using the peptide “CLEITPP(pS)SEKL” as an immunogen. The Myc and HA monoclonal antibodies were obtained from Sigma. An anti-β-tubulin mouse monoclonal antibody was purchased from Santa Cruz Biotechnology. A protease inhibitor cocktail was procured from Roche Applied Science. The cell culture reagents were obtained from Invitrogen. Enhanced chemiluminescence (ECL) detection kits were purchased from GE Healthcare. Horseradish peroxidase (HRP)-conjugated anti-mouse and anti-rabbit IgG were obtained from Invitrogen.

### Construction of truncated Odf2 and cell transfection

Full-length Odf2 and 6 truncated Odf2 sequences were amplified from murine testis cDNA via PCR and ligated into the pCMV-HA vector in frame. All of the constructs were verified through DNA sequencing. The sequences of full-length Odf2 and the truncated constructs were amplified with primer pairs as showed in Table 1.

Each primer contains a restriction enzyme recognition sequence, including a 5′ EcoRI sequence and a 3′ Xhol sequence, and the PCR products were cloned into the EcoRI/Xhol-digested pCMV-HA vector. Mutants of Odf2 (from serine/tyrosine to alanine) were generated through PCR mutagenesis using the TaKaRa MutanBEST Kit according to the manufacturer’s instructions.

For transfection, HEK-293T cells were cultured in Dulbecco’s Modified Eagle’s Medium containing 10% fetal calf serum (FCS). Transient transfection was performed using Lipofectamine 2000 according to the manufacturer’s instructions. The subsequent Western blot assay was described previously^21^,^23^,^24^.

### SDS-PAGE and Western blot

Cells were harvested and incubated in cell lysis buffer (20 mM Tris–HCl, pH 7.5; 150 mM NaCl; 1 mM Na_2_EDTA; 1 mM EGTA; 1% Triton; 2.5 mM sodium pyrophosphate; 1 mM Na_3_VO_4_; 1 mg/mL leupeptin) for 30 min at 4 °C with rocking. Following centrifugation (12,000 rpm) for 20 min at 4 °C, supernatants were collected, and the protein content was measured using a bicinchoninic acid (BCA) protein assay. The samples were mixed with loading buffer with 2-mercaptoethanol before being heated to 95 °C for 10 min.

Sperm lysates were prepared from the cauda epididymides of adult mice. In brief, mouse epididymides were dissected, isolated and cut at various points with a razor blade. The treated tissue was placed in 1 ml of phosphate-buffered saline (PBS) and incubated for 30 min at 37 °C. Then, the sperm in the supernatant were centrifuged at 1000 g for 5 min. The collected sperm were then resuspended in PBS and passed through an 80-mm pore size filter for purification. Sperm were directly mixed with 1× loading buffer and boiled at 95 °C for 10 min.

Cell or sperm extracts were subjected to 10% SDS-PAGE and then transferred to polyvinylidene difluoride (PVDF) membranes. The membranes were blocked in 5% non-fat milk in Tris-buffered saline (TBS) for 1 h at room temperature and then incubated with corresponding primary antibodies overnight at 4 °C with rocking, followed by washing three times in TBST (0.1% tween-20) and incubation of the corresponding horseradish peroxidase (HRP)-conjugated secondary antibody at room temperature for 1.5 h. After washing three times in TBST, the signals were developed using ECL chemiluminescence reagents on X-film.

### Animal care and use

Mice (C57BL-6 strain) were handled following the guidelines for animal care and use in research of the National Institutes of Health, and all animal-related procedures were approved by the State Key Laboratory of Genetic Engineering and Institutional Animal Care and Use Committee (IACUC) of Fudan University. Mouse breeding and animal-related protocols were performed following the general guidelines published by the Association for Assessment and Accreditation of Laboratory Animal Care (AAALAC).

### Phosphopeptide enrichment and identification

Gel slices containing the Odf2 protein were de-stained with 50% acetonitrile (ACN) in 50 mM ammonium bicarbonate. The dried gel slices were sequentially reduced with 10 mM DTT and alkylated with 55-mM iodoacetamide. The proteins were then digested overnight at 37 °C with sequencing-grade modified trypsin at a protein-to-enzyme ratio of 50:1. The peptides were extracted from the gel slices with 60% ACN and 1% trifluoroacetic acid (TFA) and were then desalted with a MicroTrap C8. Phosphopeptides were subsequently enriched with a MicroTrap Titanium Dioxide Phosphopeptide Trap (1 × 8 mm, Michrom), as described in the manual. Briefly, the reaction products dissolved in equilibration buffer (2% ACN, 5% TFA) were loaded into the pre-equilibrated TiO_2_ MicroTrap, followed by washing with 80% ACN and 5% TFA and subsequent elution with 2% ACN and 1% NH_4_OH. The eluted phosphopeptides were desalted with a MicroTrap C8 (Michrom, US) and lyophilized using a SpeedVac (ThermoSavant, USA), then resuspended in 20 μl of 0.1% formic acid/2% acetonitrile. All mass spectrometric experiments were performed on a LTQ orbitrap “XL” mass spectrometer connected to a Paradigm MDLC nanoflow LC system via an ADVANCE Spray Source LC-MS interface (MICHROM Bioresources, USA). The peptide mixture was loaded onto a 15 cm column with a 0.1 mm inner diameter packed with Magic C18AQ 3-um Reversed Phase resins and separated through a 60 minute linear gradient from 100% solvent A (0.1% formic acid/5% acetonitrile/95% water) to 35% solvent B (0.1% formic acid/100% acetonitrile) at a flow rate of 500 nl/min. The spray voltage was set to 1.5 KV, and the temperature of the ion transfer capillary was 160 °C. The mass spectrometer was operated in positive ion mode and used in data-dependent mode to automatically switch between MS and MS/MS using the Tune and Xcalibur 2.5.5 software package. One full MS scan from 350 to 1800 m/z was acquired at a high resolution of R = 100,000 (defined at m/z = 400), followed by 10 data = dependent MS/MS spectra in the linear ion trap from the 10 most abundant ions. All MS/MS ion spectra were analyzed using Sequest1 (Thermo Fisher Scientific, San Jose, CA, USA; version v.27, rev. 11) and were incorporated into the Sorcerer engine version 4.0.4 build (Sage-N Research). Sequest was set up to search the ipi.MOUSE.v3.65 database (56775 entries) (ftp.ebi.ac.uk/pub/databases/IPI/current) assuming semi-enzyme tryptic digestion allowing 3 missed tryptic cleavages, with a full mass from 600 to 4600. The precursor ion tolerance was set to ±10 p.p.m, and the MS2 ion tolerance was set to 1 Da. The search parameters for the total peptides allowed a static modification of Carbamidomethyl C (+57.021465) on cysteine and a dynamic modification of oxidized M (+15.99492) on methionine. Additional searches for phosphopeptides allowed dynamic modifications of phosphorylation STY (+79.966331), on serine, threonine, and tyrosine, with up to 6 total dynamic modifications and up to 4 of a particular dynamic modification. The cutoff values of the filter threshold were set at XCorr 2+, 3+, 4+: >2.5, >3.0, >3.5 and DeltaCN > 0.08, and two peptides were required for total peptide to protein identification, with an estimated false-discovery rate (FDR) < 1%. Finally, the results were presented using the Scaffold viewer.

## Additional Information

**How to cite this article**: Wang, X. *et al.* Testis-specific serine/threonine protein kinase 4 (Tssk4) phosphorylates Odf2 at Ser-76. *Sci. Rep.* **6**, 22861; doi: 10.1038/srep22861 (2016).

## Figures and Tables

**Table 1:** Primer sequences for Odf2 truncated constructs.

Primer name	Primer sequences
Odf2-FL-F	5′-GATGAATTCGGATGTCTGCCTCATCCTCAGGCG-3′
Odf2-FL-R	5′-GATCTCGAGTCATCTCGGTAAGCGGGCCCCTG-3′
Odf2-C1-F	5′-GATGAATTCGGTCTACAGAAGACGACGATTCAG-3′
Odf2-C1-R	5′-GATCTCGAGTCATCTCGGTAAGCGGGCCCCTG-3′
Odf2-C2-F	5′-GATGAATTCGGGGGAAGCTGAAAACCGAGAAAC-3′
Odf2-C2-R	5′-GATCTCGAGTCATCTCGGTAAGCGGGCCCCTG-3′
Odf2-C3-F	5′-GATGAATTCGGCGAGACAAAGAGACCCTAAAG-3′
Odf2-C3-R	5′-GATCTCGAGTCATCTCGGTAAGCGGGCCCCTG-3′
Odf2-N-F	5′-GATGAATTCGGATGTCTGCCTCATCCTCAGGCG-3′
Odf2-N-R	5′-GATCTCGAGGATGGTATCCTTCAAGGCCATG-3′
Odf2-M1-F	5′-GATGAATTCGGTCTACAGAAGACGACGATTCAG-3′
Odf2-M1-R	5′-GATCTCGAGGATGGTATCCTTCAAGGCCATG-3′
Odf2-M2-F	5′-GATGAATTCGGGGGAAGCTGAAAACCGAGAAAC-3′
Odf2-M2-R	5′-GATCTCGAGGTCTCCCTTCTGCTTCAGTTCC-3′
